# Immunity to *Lutzomyia intermedia* Saliva Modulates the Inflammatory Environment Induced by *Leishmania braziliensis*

**DOI:** 10.1371/journal.pntd.0000712

**Published:** 2010-06-15

**Authors:** Tatiana R. de Moura, Fabiano Oliveira, Gabriele C. Rodrigues, Marcia W. Carneiro, Kiyoshi F. Fukutani, Fernanda O. Novais, José Carlos Miranda, Manoel Barral-Netto, Claudia Brodskyn, Aldina Barral, Camila I. de Oliveira

**Affiliations:** 1 Centro de Pesquisas Gonçalo Moniz, Fundação Oswaldo Cruz (FIOCRUZ), Salvador, Brazil; 2 Vector Molecular Biology Unit, National Institute of Allergy and Infectious Diseases, National Institutes of Health, Bethesda, Maryland, United States of America; 3 Universidade Federal da Bahia, Salvador, Brazil; 4 Instituto Nacional de Ciência e Tecnologia (INCT) de Investigação em Imunologia, São Paulo, Brazil; Institut Pasteur, France

## Abstract

**Background:**

During blood feeding, sand flies inject *Leishmania* parasites in the presence of saliva. The types and functions of cells present at the first host-parasite contact are critical to the outcome on infection and sand fly saliva has been shown to play an important role in this setting. Herein, we investigated the *in vivo* chemotactic effects of *Lutzomyia intermedia* saliva, the vector of *Leishmania braziliensis*, combined or not with the parasite.

**Methods and Findings:**

We tested the initial response induced by *Lutzomyia intermedia* salivary gland sonicate (SGS) in BALB/c mice employing the air pouch model of inflammation. *L. intermedia* SGS induced a rapid influx of macrophages and neutrophils. In mice that were pre-sensitized with *L. intermedia* saliva, injection of SGS was associated with increased neutrophil recruitment and a significant up-regulation of CXCL1, CCL2, CCL4 and TNF-α expression. Surprisingly, in mice that were pre-exposed to SGS, a combination of SGS and *L. braziliensis* induced a significant migration of neutrophils and an important modulation in cytokine and chemokine expression as shown by decreased CXCL10 expression and increased IL-10 expression.

**Conclusion:**

These results confirm that sand fly saliva modulates the initial host response. More importantly, pre-exposure to *L. intermedia* saliva significantly modifies the host's response to *L. braziliensis*, in terms of cellular recruitment and expression of cytokines and chemokines. This particular immune modulation may, in turn, favor parasite multiplication.

## Author Summary

Transmission of *Leishmania* parasites occurs during blood feeding, when infected female sand flies inject humans with parasites and saliva. Chemokines and cytokines are secreted proteins that regulate the initial immune responses and have the potential of attracting and activating cells. Herein, we studied the expression of such molecules and the cellular recruitment induced by salivary proteins of the *Lutzomyia intermedia* sand fly. Of note, *Lutzomyia intermedia* is the main vector of *Leishmania braziliensis*, a parasite species that causes cutaneous leishmaniasis, a disease associated with the development of destructive skin lesions that can be fatal if left untreated. We observed that *L. intermedia* salivary proteins induce a potent cellular recruitment and modify the expression profile of chemokines and cytokines in mice. More importantly, in mice previously immunized with *L. intermedia* saliva, the alteration in the initial inflammatory response was even more pronounced, in terms of the number of cells recruited and in terms of gene expression pattern. These findings indicate that an existing immunity to *L. intermedia* sand fly induces an important modulation in the initial immune response that may, in turn, promote parasite multiplication, leading to the development of cutaneous leishmaniasis.

## Introduction

The intracellular protozoan parasites of the *Leishmania* species are transmitted to vertebrate host through the bites of sand flies. Within the vertebrate host, *Leishmania* parasites reside in phagocytes and induce a spectrum of diseases ranging from a single self-healing cutaneous lesion to the lethal visceral form. It is currently estimated that leishmaniasis affects two million people per year worldwide [1].

*Leishmania braziliensis*, the main causative agent of cutaneous leishmaniasis (CL) in Brazil, can be transmitted to the human host by the bite of the sand fly *Lutzomyia intermedia*. [2], [3]. Several studies have shown that pre-exposure to saliva or to bites from uninfected sand flies results in protection against subsequent infection with *Leishmania major* [4]–[7], *Leishmania. amazonensis* [8], and *Leishmania chagasi* [9]. On the contrary, pre-exposure to *Lutzomyia intermedia* saliva enhanced infection with *L. braziliensis* in the mouse model; disease exacerbation was correlated with generation of a Th2 response evidenced by a reduction in the IFN-γ/IL-4 ratio [10]. Importantly, individuals with active CL showed higher humoral immune responses to *L. intermedia* saliva compared with control subjects, a finding also demonstrated with Old World CL [11] . These data indicate an association between disease and immune response to *L. intermedia* saliva in humans.

In the case of *L. intermedia*, the lack of protection observed following pre-exposure to saliva in the murine model may be related to differences in the initial inflammatory response induced by the salivary proteins. Several studies have shown the potential of salivary antigens from *Lutzomyia longipalpis*, *Phlebotomus duboscqi*, *Phlebotomus papatasi* and *Phlebotomus ariasi* to modulate cell recruitment and production of immune response mediators [12]–[17] however, little is known regarding these effects when using *L. intermedia* saliva. Our group has previously shown that pre-treatment of human monocytes with *L. intermedia* followed by *L. braziliensis* infection led to a significant increase in TNF-α, IL-6, and IL-8 production [18], indicating the ability of *L. intermedia* saliva to alter the inflammatory milieu. To gain further information regarding the events associated with the initial host response to *L. intermedia* saliva, we employed the air pouch model of inflammation. This model simulates inoculation of the sand fly in a closed environment and allows for subsequent analysis of inflammatory parameters and mediators induced in vivo by distinct stimuli [19]. Using this model, we showed that saliva from *L. longipalpis* rapidly induced CCL2 expression and macrophage recruitment, in synergy with *L. chagasi* parasites, in BALB/c mice [20]. Here we describe the ability of *L. intermedia* salivary gland sonicate (SGS) to modulate the host immune response in naïve and in SGS-sensitized mice. We have demonstrated that *L. intermedia* salivary proteins induce neutrophil recruitment and modulate cytokine and chemokine expression. Crucially, a downregulation in CXCL10 paralleled by an increase in IL-10 expression was observed in SGS-sensitized mice stimulated with saliva+*L. braziliensis*. This correlates with disease exacerbation previously observed in mice immune to *L. intermedia* SGS and challenged with *L. braziliensis* [10].

## Methods

### Parasite culture

*Leishmania braziliensis* promastigotes (strain MHOM/BR/01/BA788 [21]) were grown in Schneider medium (Sigma Chemical Corporation, St. Louis, MO, USA) supplemented with 100 U/ml of penicillin, 100 µg/ml of streptomycin, 10% heat-inactivated fetal calf serum (all from Invitrogen, San Diego, CA, USA), and 2% sterile human urine. Stationary-phase promastigotes from second passage culture were used in all experiments.

### Mice

Female BALB/c mice (6–8 weeks of age) were obtained from CPqGM/FIOCRUZ Animal Facility where they were maintained under pathogen-free conditions. All procedures involving animals were approved by the local Ethics Committee on Animal Care and Utilization (CEUA—CPqGM/FIOCRUZ).

### Sand flies and preparation of SGS

Adult *Lutzomyia intermedia* sand flies were captured in Corte de Pedra, Bahia, and were used for dissection of salivary glands. Salivary glands were stored in groups of 20 pairs in 20 µl NaCl (150 mM)-Hepes buffer (10 mM; pH7.4) at −70°C. Immediately before use, salivary glands were disrupted by ultrasonication in 1.5-ml conical tubes. Tubes were centrifuged at 10,000×g for two minutes, and the resultant supernatant—salivary gland sonicate (SGS)—was used for the studies. The level of lipopolysaccharide (LPS) contamination of SGS preparations was determined using a commercially available LAL chromogenic kit (QCL-1000; Lonza Biologics, Portsmouth, NH, USA); LPS concentration was <0.1 ng/ml.

### Sand fly saliva immunization

BALB/c mice (groups of five to six) were immunized three times with SGS (equivalent to one pair of salivary glands) in 10 µl of PBS in the dermis of the right ear using a 27.5 G needle. Immunizations were performed at two-week intervals. Control mice were injected with PBS. Development of an immune response against *L. intermedia* saliva was confirmed by ELISA as previously described [10].Immune sera were pooled from SGS-immunized mice and employed in neutralization experiments. Immune mice were employed in air pouch experiments.

### In vivo cell recruitment into the air pouch

Air pouches were raised on the dorsum of anesthetized BALB/c mice (groups of five to six) by injection of 3 ml of air, as described elsewhere [22]. Air pouches were inoculated with either one of the following stimuli: *L. intermedia* SGS (equivalent to one pair of salivary glands/animal); *L. intermedia* SGS pre-incubated with a pool of anti-SGS immune sera (SGS+50 µl of immune serum pre-incubated for one hour at 37°C); a pool of anti-SGS immune sera alone; stationary-phase *L. braziliensis* promastigotes (10^5^ parasites); or *L. braziliensis*+SGS. Air pouches in control mice were injected with endotoxin-free saline (negative control) or with LPS (Calbiochem, San Diego, CA, USA) (20 µg/ml; positive control). After twelve hours, animals were euthanized and pouches washed with 5 ml of endotoxin-free saline for collection of exudates containing leukocytes. Lavage fluids were washed, and cell pellets were resuspended in saline, stained in Turk's solution, and counted in a Neubauer hemocytometer. Cells were cytoadhered to glass slides using Shandon cytospin2 and stained with hematoxylin and eosin to determine proportions of monocytes/macrophages, neutrophils, lymphocytes, basophils, and eosinophils. Air pouch lining tissue was placed in 5–10 volumes of RNAlater (Ambion Inc., Austin, TX, USA), and samples were stored at −80°C.

### RNA isolation and real-time PCR

Total RNA was extracted from the air pouch lining tissue using the RNeasy Protect Mini Kit (Qiagen, Inc., Santa Clara, CA, USA) according to manufacturer's instructions. The resulting RNA was resuspended in 20 µl diethyl pyrocarbonate (DEPC)-treated water and stored at −80°C until use. cDNA synthesis for detection of cytokine mRNA was performed after reverse transcription (Im Prom-II™ reverse transcription system). Real-time PCR was performed in triplicate on the Abi Prism 7500 (Applied Biosystems, Inc., Fullerton, CA, USA); thermal cycle conditions consisted of a two-minute initial incubation at 50°C followed by ten-minute denaturation at 95°C and 50 cycles at 95°C for 15 seconds and 60°C for one minute each. Each sample and the negative control were analyzed in triplicate for each run. The comparative method was used to analyze gene expression. Chemokine or cytokine cycle threshold (C_t_) values were normalized to GAPDH expression as determined by ΔC_t_ = C_t (target gene)_−C_t (GAPDH gene)_. Fold change was determined by 2^−ΔΔCt^, where ΔΔC_t_ = ΔC_t (target)_−ΔC_t (saline)_ [23]. The following primers were employed: GAPDH (Forward: 5′-TGTGTCCGTCGTGGATCT GA-3′; Reverse: 5′-CCTGCTTCACCACCTTCTTGA-3′); CCL2 (Forward: 5′-CAGGTC CCTGTCATGCTTCTG-3′; Reverse: 5′-GAGCCAACACGTGGATGCT-3′) ; CCL3 (Forward: 5′-TCTTCTCAGCGCCATATGGA-3′; Reverse: 5′-CGTGGAATCTTCCGG CTGTA-3′); CCL4 (Forward: 5′-TGCTCGTGGCTGCCTTCT-3′; Reverse: 5′-CAGGAA GTGGGAGGGTCAGA-3′); CXCL1: (Forward: 5′-CCGAAGTCATAGCCACACTCAA-3′; Reverse: 5′-AATTTTCTGAACCAAGGGAGCTT-3′); CXCL10: (Forward: 5′-GGACGG TCCGCTGCAA-3′; Reverse: 5′-CCCTATGGCCCTCATTCTCA-3′); IFN-γ (Forward: 5′-CTACACACTGCATCTTGGCTTTG-3′; Reverse: 5′-TGACTGCGTGGCAGTA-3′); TNF-α (Forward: 5′-GGTCCCCAAAGGGATGAGAA-3′; Reverse: 5′-TGAGGGTCT GGGCCATAGAA-3′); and IL-10 (Forward: 5′-CAGCCGGGAAGACAATAACTG-3′; Reverse: 5′-CGCAGCTCTAGGAGCATGTG-3′). Primers were designed using Primer Express Software (Applied Biosystems).

### Histology and immunohistochemistry

BALB/c mice (n = 5) were intradermally immunized with *L. intermedia* SGS (equivalent to one pair of salivary glands) or injected with PBS three times in the right ear at two-week intervals. After the third injection, pre-sensitized or control animals were intradermally inoculated with *L. intermedia* SGS, in the opposite (left) ear dermis. Twenty-four and forty-eight hours after SGS injection, animals were euthanized and the ear was biopsied and stored in 10% neutral buffered formalin. Ears were mounted in paraffin blocks, sectioned at 5-µm intervals, and stained with hematoxylin and eosin for histologic analysis. Paraffin-embedded sections of ears fixed in 10% neutral buffered formalin were used for immunohistochemistry. Myeloperoxidase rabbit anti-mouse (Dako, Carpenteria, CA, USA) was used at 1∶1000 dilution. A secondary biotinylated goat anti-rabbit antibody was used at 1∶500 for 15 minutes (Vector Laboratories, Burlingame, CA, USA) and detected by R.T.U. Vectastin Elite ABC reagent (Vector Laboratories) and DAB chromagen.

### Statistical analysis

Data are presented as the mean with 95%CI. The significance of the results was calculated using nonparametric statistical tests: two-sided Mann-Whitney for comparisons between two groups; Kruskal-Wallis followed by Dunn's multiple comparison test for comparisons between three groups. Analyses were conducted using Prism (GraphPad Software Inc., San Diego, CA, USA); a P-value of <0.05 was considered significant.

## Results

### In vivo effect of *L. intermedia* SGS on leukocyte recruitment

**Figure 1:**
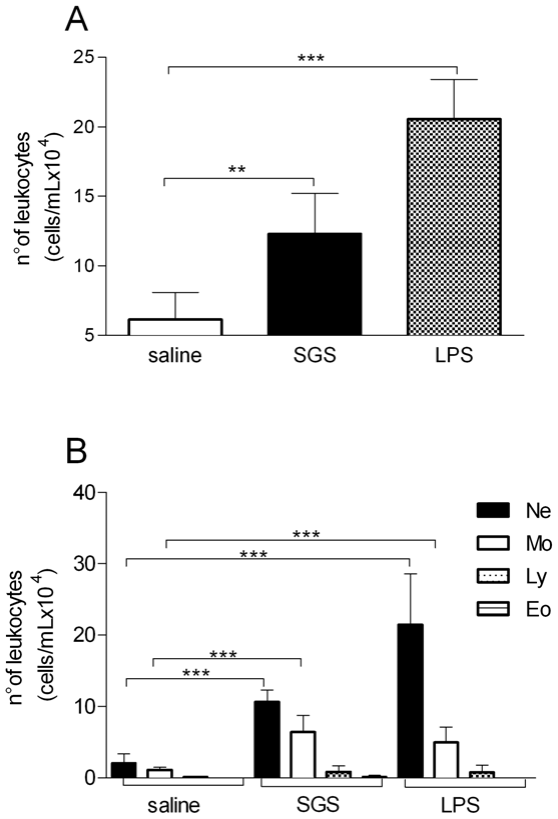
Leukocyte recruitment in air pouch exudates in response to *L. intermedia* saliva. Air pouches were raised on BALB/c mice (five to six per group) and were inoculated with either endotoxin-free saline, *L. intermedia* SGS, or LPS. Exudates were collected twelve hours later. Leukocytes were enumerated microscopically. (A) Total number of leukocytes and (B) total number of neutrophils, monocytes, eosinophils, and lymphocytes accumulated in air pouches. The data are representative of three independent experiments. (** P<0.01; *** P<0.001).

We initially studied the cellular recruitment induced by *L. intermedia* SGS inoculation. Air pouches were induced in BALB/c mice and subsequently probed with different stimuli: endotoxin-free saline; *L. intermedia* SGS; or LPS. *L. intermedia* SGS induced a significant increase in leukocyte accumulation in the air pouch compared with saline injection (Figure. 1A). Most cells recruited by inoculation of *L. intermedia* SGS into air pouches were neutrophils, followed by monocytes (Figure 1B). LPS inoculation was used as a positive control for cell recruitment and, as expected, led to a predominant recruitment of neutrophils (Figure 1B). Moreover, inoculation of *L. intermedia* SGS did not lead to significant changes in either eosinophil or lymphocyte recruitment.

### Anti-SGS antibodies inhibit leukocyte recruitment induced by *L. intermedia* SGS

**Figure 2:**
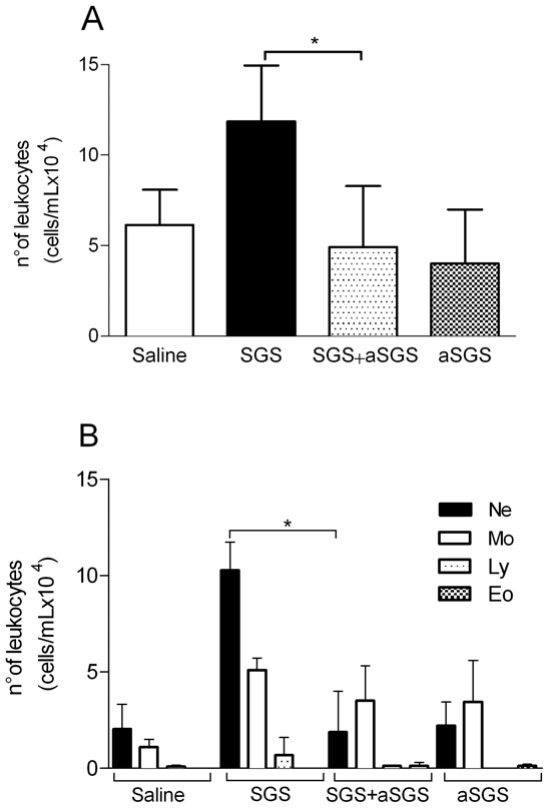
Pre-incubation of *L. intermedia* saliva with immune serum inhibits leukocyte recruitment. Air pouches were raised on BALB/c mice (five to six per group) and were inoculated with either endotoxin-free saline, *L. intermedia* SGS, *L. intermedia* SGS+immune sera (SGS+α-SGS), or immune sera alone (α-SGS). Exudates were collected twelve hours after initial stimulation. Leukocytes were enumerated microscopically. (A) Total number of leukocytes and (B) total number of neutrophils, monocytes, eosinophils, and lymphocytes accumulated in air pouches. The data shown are from a single experiment representative of three independent experiments. (* P<0.05; ** P<0.01).

To confirm that the effect of *L. intermedia* SGS on leukocyte accumulation within air pouches was specific, we pre-incubated SGS with anti-SGS immune sera obtained from mice immunized with *L. intermedia* SGS (data not shown, [10] ). Pre-incubation of *L. intermedia* SGS with anti-SGS immune sera inhibited leukocyte accumulation induced by *L. intermedia* SGS by 56% (Figure 2A), whereas air-pouch inoculation with immune sera alone led to a cellular recruitment similar to that induced by saline (Figure 2A). Notably, the significant decrease in cellular recruitment following incubation of *L. intermedia* SGS with antisera was associated with a significant reduction (81%) in the number accumulating neutrophils (Figure 2B). Recruitment of monocytes, lymphocytes, and eosinophils, however, remained unchanged (Figure 2B).

### Enhanced neutrophil recruitment in mice immunized with *L. intermedia* saliva

**Figure 3:**
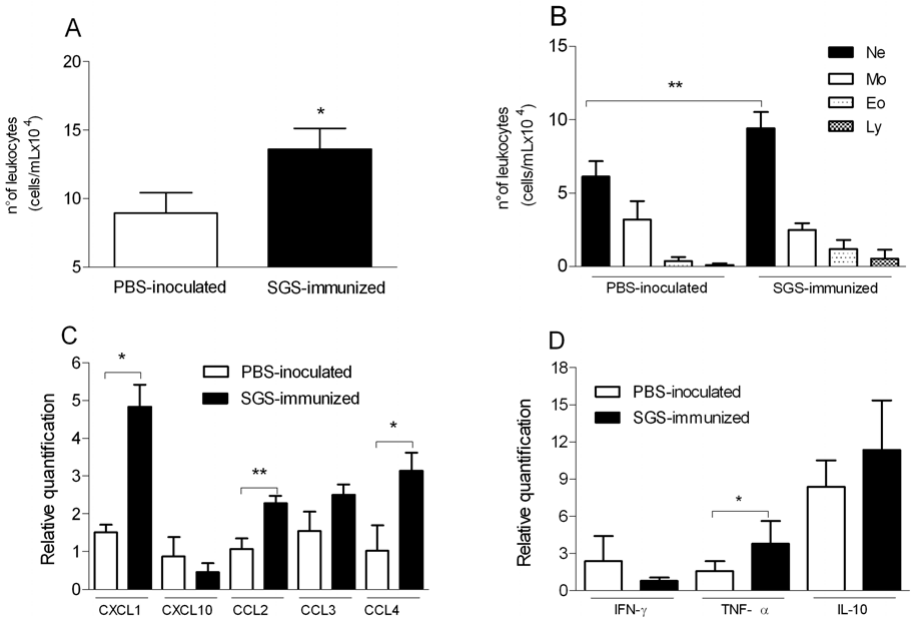
Modulation in leukocyte recruitment and gene expression in mice pre-exposed to *L. intermedia* saliva. BALB/c mice (five to six per group) received three inoculations with endotoxin-free PBS or *L. intermedia* SGS. Fifteen days after the last immunization, air pouches were raised and inoculated with *L. intermedia* SGS. Exudates and air pouch lining tissue were collected twelve hours later. Leukocytes were enumerated microscopically. (A) Total number of recruited leukocyte and (B) total number of neutrophils, monocytes, eosinophils, and lymphocytes accumulated in air pouches. Relative expression of chemokines (C) and cytokines (D) in the air pouch lining tissue was determined by real-time PCR. The data shown are from a single experiment representative of two independent experiments. (* P<0.05; ** P<0.01).

*L. intermedia* SGS was able to induce a significant increase in leukocyte recruitment in the air-pouch model of inflammation when compared with saline (Figure 1). This effect was particularly powerful on neutrophil migration and was abrogated when SGS was pre-incubated with anti-SGS-specific antiserum (Figure 2B). We then investigated the initial inflammatory response in mice that had been previously immunized with *L. intermedia* SGS. Air pouches were raised on the back of immune mice, and pouches were stimulated with *L. intermedia* SGS. Control mice were injected with endotoxin-free PBS. Mice immunized with *L. intermedia* SGS showed a significant increase in the total number of leukocytes (Figure 3A) accumulating in the air pouch compared with control mice injected with PBS. Surprisingly, this increase was associated with an accumulation of neutrophils (53%) migrating to the air pouch (Figure 3B), whereas migration of monocytes, eosinophils, and lymphocytes remained unaltered in SGS-immunized mice compared with control mice injected with PBS. Because chemokines, together with adhesion molecules, are key controllers of leukocyte migration, we tested for chemokine expression in the pouch lining tissue. CXC-class chemokines act mainly on neutrophils, whereas CC-class chemokines act on a larger group of cells including monocytes, eosinophils, and lymphocytes. Additionally, cytokines have long been recognized as key elements in the host response against *Leishmania* (reviewed in [24]. As shown in Figure 3C, expression of CXCL1, CCL2, and CCL4 was significantly upregulated in SGS-immunized mice compared with control mice injected with PBS. Moreover, SGS-immune mice also displayed a significant increase in TNF-α expression without significant modulation in expression of IL-10 or IFN-γ (Figure 3D).

**Figure 4:**
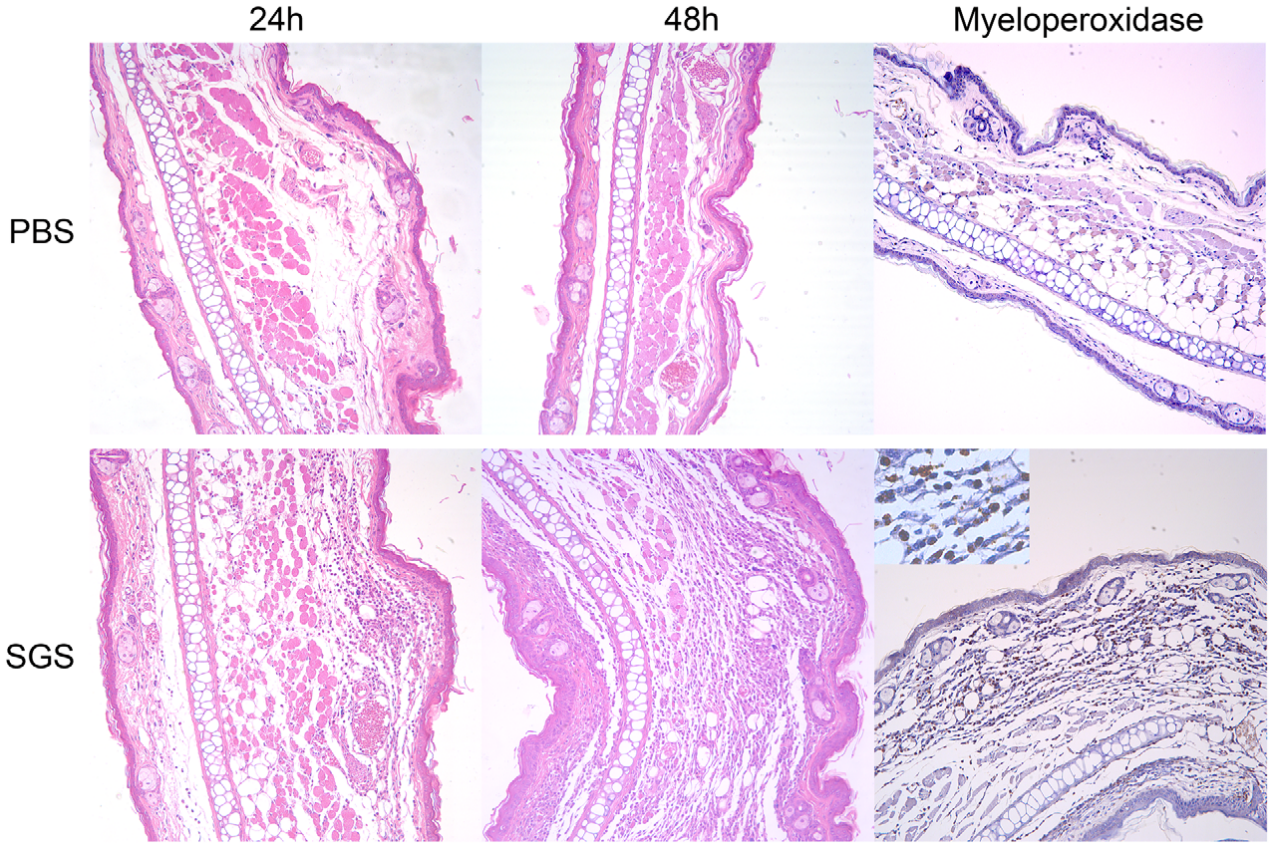
Enhanced neutrophil recruitment the ear dermis of mice immunized with *L. intermedia* saliva. Ears of BALB/c mice (five per group) were injected with PBS (control) or were immunized with *L. intermedia* SGS (five per group). Both groups were challenged in the contra lateral ear with *L. intermedia* SGS. Ear sections were obtained twenty-four and forty-eight hours after challenge and stained with H&E. Neutrophils were detected by myeloperoxidase staining at forty-eight hours after challenge with SGS. Sections were analyzed by optical microscopy under 200× and 400× magnifications (insert). Sections from one representative experiment are shown.

We then investigated whether the neutrophil accumulation effect observed in air pouches raised in SGS-immune mice and stimulated with SGS could be replicated in the ear dermis. As shown in Figure 4, ear sections from control mice injected with PBS showed very few inflammatory cells at either 24 or 48 hours after SGS challenge. In contrast, ear sections from SGS-immunized mice displayed, 24 hours after SGS-challenge, numerous polymorphonuclear and few mononuclear cells (Figure 4); at 48 hours, the inflammatory infiltrate was further increased. Presence of neutrophils was confirmed by myeloperoxidase staining and was not observed in control mice injected with PBS.

### In vivo effect of *L. intermedia* SGS on leukocyte recruitment induced by *L. braziliens*is alone or in combination with SGS

**Figure 5:**
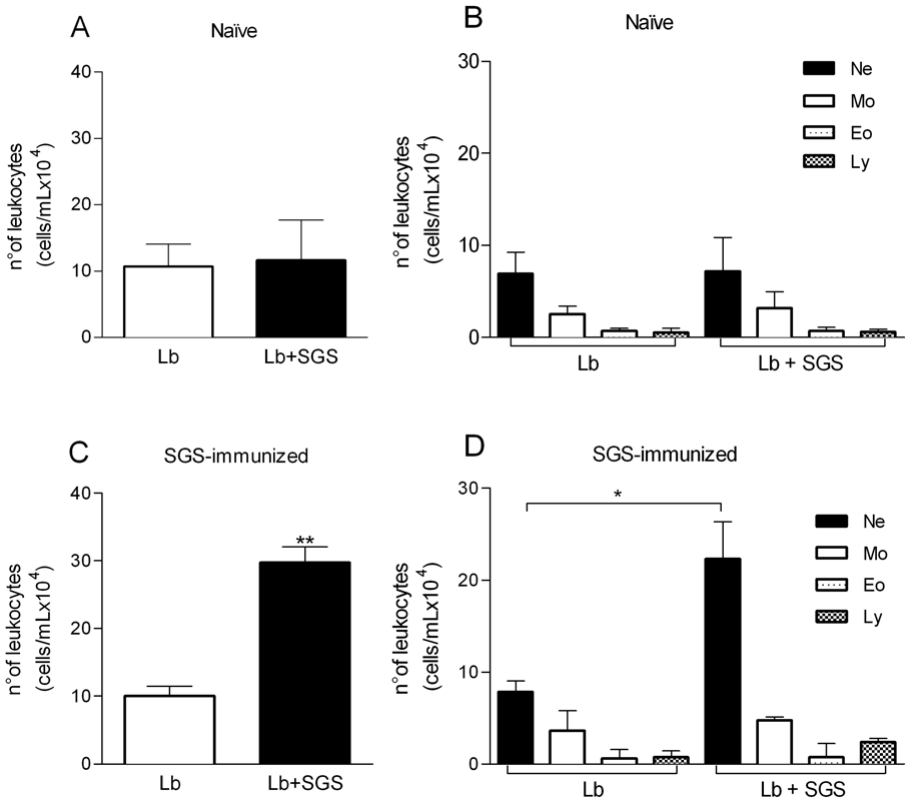
Leukocyte recruitment in air pouch exudates in response to *L. braziliensis* parasites and *L. intermedia* saliva. BALB/c mice (five to six per group) received three immunizations with *L. intermedia* SGS. Fifteen days after the last immunization, air pouches were raised in naïve mice and in mice immunized with *L. intermedia* SGS. Air pouches were inoculated with *L. braziliensis* alone (Lb) or with *L. braziliensis*+*L. intermedia* saliva (Lb+SGS). Exudates were collected twelve hours later. Leukocytes were enumerated microscopically. (A) Total number of leukocytes accumulated in air pouches and (B) total number of neutrophils, monocytes, eosinophils, and lymphocytes accumulated in air pouches of naïve mice. (C) Total number of leukocytes accumulated in air pouches and (D) total number of neutrophils, monocytes, eosinophils, and lymphocytes accumulated in air pouches of SGS-immunized mice The data shown are from a single experiment representative of three independent experiments. (* P<0.05).

Because SGS-immune mice displayed enhanced neutrophil recruitment, we investigated whether the presence of *L. braziliensis*, the parasite transmitted by *L. intermedia* sand flies, would exert any effect in this outcome. Therefore, air pouches were raised on the back of either naïve or SGS-immunized mice and pouches were stimulated with *L. braziliensis* (Lb) or *L. braziliensis*+*L. intermedia* SGS (Lb+SGS). In naïve mice, we did not detect significant differences in the number of accumulating leukocytes (Figure 5A) or in the recruited cell subsets (Figure 5B) following inoculation with Lb or Lb+SGS (Figure 5B). On the contrary, in SGS-immunized mice, Lb+SGS led to a robust and significant increase in the number of accumulating leukocytes compared with Lb alone (Figure 5C). The increase in the number of leukocytes was due to accumulation of neutrophils in the pouches upon inoculation of Lb+SGS (Figure 5D). There was no significant modulation in the recruitment of monocytes, eosinophils, or lymphocytes in naïve or SGS-immunized mice upon inoculation of Lb or Lb+SGS (Figure 5B and 5D, respectively).

### In vivo effect of *L. intermedia* SGS on chemokine and cytokine expression induced by *L. braziliensis* alone or in combination with SGS

**Figure 6:**
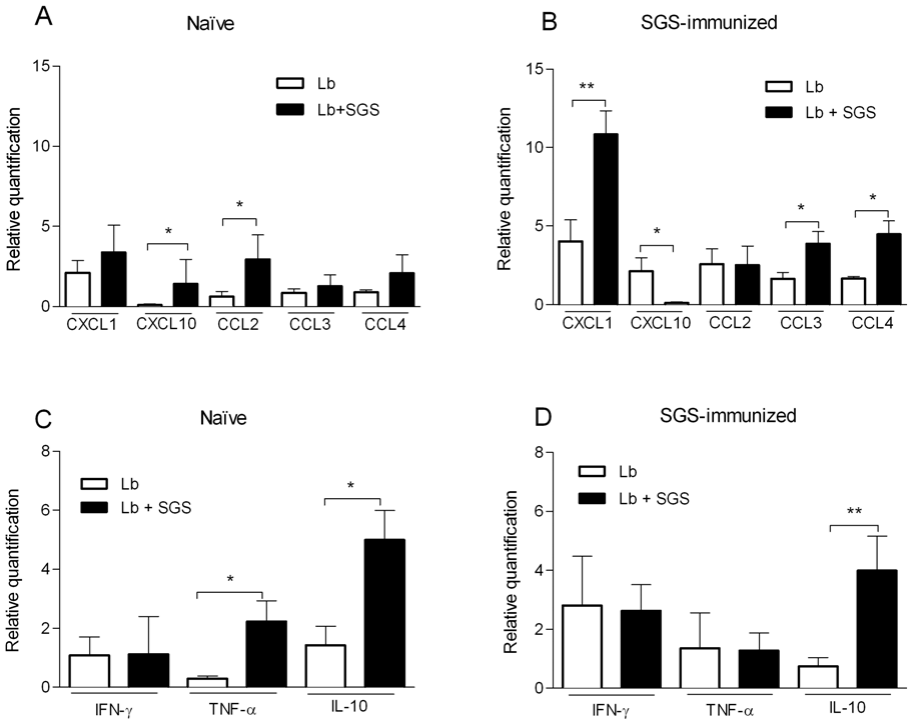
Chemokine and cytokine expression in air pouch exudates in response to *L. braziliensis* parasites and *L. intermedia* saliva. BALB/c mice (five to six per group) received three immunizations with *L. intermedia* SGS. Fifteen days after the last immunization, air pouches were raised in naïve mice and mice immunized with *L. intermedia* SGS. Air pouches were inoculated with *L. braziliensis* alone (Lb) or with *L. braziliensis*+*L. intermedia* saliva (Lb+SGS). Air pouch lining tissue was submitted to real-time PCR for relative quantification of chemokines and cytokines. Chemokine expression in (A) naïve and (B) SGS-immunized mice. Cytokine expression in (C) naïve and (D) SGS-immunized mice. Bars represent the means and standard errors of the means of five mice per group. The data shown are from a single experiment representative of two independent experiments. (* P<0.05; ** P<0.01).

We then investigated the modulation in cytokine and chemokine expression induced by *L. braziliensis* alone or in the presence of saliva in naïve and in SGS-immunized mice. In naïve mice, pouch stimulation with Lb+SGS induced a significant increase in CXCL10 and CCL2 expression compared with pouch inoculation with Lb alone (Figure 6A). In SGS-immunized mice, chemokine expression was over two-fold higher compared with naïve mice (Figure 6B). More important, pouch inoculation with Lb+SGS led to a different pattern of chemokine expression as indicated by a significant upregulation in expression of CXCL1, CCL3, and CCL4 compared with inoculation of Lb alone (Figure 6B). Of note, in SGS-immunized mice, pouch inoculation with Lb+SGS led to a significant decrease in CXCL10 expression (Figure 6B) as opposed to naïve mice, in which pouch inoculation with Lb+SGS led to upregulation in CXCL10 expression (Figure 6A). Regarding cytokine expression, naïve mice displayed augmented expression of both TNF-α and IL-10 upon pouch inoculation with Lb+SGS (Figure 6C) compared with inoculation with Lb alone. In SGS-immunized mice, stimulation with Lb+SGS led to specific increase in IL-10 expression (Figure 6D). In this same group, inoculation of Lb+SGS was not capable of significantly decreasing expression of IFN-γ and TNF-α (Figure 6D).

## Discussion

Sand flies use saliva to manipulate host homoeostasis, favoring the acquisition of a blood meal. These sand fly salivary molecules modify the skin microenvironment and this, in turn, may favor infection by *Leishmania* parasites (rev. in [25]). Indeed, we previously observed that *L. intermedia* SGS-immune mice show a higher disease burden when challenged with *L. braziliensis* [10]. To gain understanding of the early events associated with inoculation of *L. intermedia* sand fly saliva, we evaluated leukocyte migration and chemokine/cytokine expression induced in the air-pouch model of inflammation. Importantly, the *L. intermedia* sand fly is the vector of *L. braziliensis* [2], [3], the main etiologic agent of cutaneous leishmaniasis.

Injection of *L. intermedia* SGS into air pouches led to a significant increase in the recruitment of neutrophils and monocytes, corroborating previous findings that both of these cell populations are recruited to the site of saliva inoculation [7], [10], [12], [17], [20], [26]. Indeed, the initial events following saliva inoculation have recently been explored by in vivo live imaging [27]. It was shown that sand fly biting leads to potent neutrophil migration and that these cells are efficiently infected by *L. major*, indicating that neutrophils may serve as host cells for *Leishmania* in the early phase of infection, as previously suggested [28], [29]. Differently from *L. longipalpis* saliva [20], *L. intermedia* did not lead to accumulation of eosinophils, which are strongly related to mosquito bites and allergies. This distinction in the cellular recruitment induced by *L. intermedia* vs. *L. longipalpis* saliva may be explained by variation in the salivary components within sand flies, such as maxadilan, present only in *L. longipalpis* [30], and hyaluronidase, present in both *L. longipalpis* and various species within the genus *Phlebotomus* [31], [32].

Pre-incubation of *L. intermedia* SGS with specific antisera was able to partially neutralize the leukocyte-recruiting effects of SGS, mainly decreasing the number of accumulating neutrophils, without a significant effect on monocytes. Similarly, Belkaid et al. showed that anti-SGS antibodies could neutralize the ability of *P. papatasi* SGS to enhance *L. major* infection in BALB/c mice [5]; however, SGS-immune mice showed an enhanced neutrophil recruitment upon stimulation with SGS in pre-sensitized animals. The actual levels of anti-saliva antibodies into the pouch exudates are unknown and may not be sufficient to neutralize the in vivo effects of the saliva. Another possibility for the in vivo findings is that salivary molecules are able to trigger cytokine/chemokine expression, despite the presence of neutralizing antibodies, leading to enhanced neutrophil recruitment.

Leukocyte recruitment to sites of inflammation is a key event in both innate and adaptive immunity, and chemokines are major players that regulate the sequential steps of leukocyte rolling, firm adherence, and transmigration. In this sense, we tested for CXC-class chemokines, that act mainly on neutrophils, and CC-class chemokines that act on a larger group of cells including monocytes, eosinophils, and lymphocytes. In mice sensitized and stimulated with *L. intermedia* SGS, we saw increased neutrophil recruitment and significant upregulation in the expression of CXCL1, CCL2, and CCL4. Indeed, CXC chemokines, such as CXCL1, are critical molecules for neutrophil recruitment [33], and CXCL1 is also a dominant chemokine in murine inflammatory responses [34]. CCL2 mediates neutrophil adherence and transmigration, a process dependent on activation of mast cells and release leukotrienes and PAF [35], and CCL4 expression has been associated with a type 1 immune response [36]. Therefore, the enhanced neutrophil chemotaxis in SGS-immunized mice may result from a concomitant upregulation in CXCL1 and CC chemokines (CCL2 and CCL4) and may be further amplified by upregulation in TNF-α, favoring a pro-inflammatory environment as shown by upregulation in CCL4 expression. Indeed, OVA-immunized mice displayed increased neutrophil migration upon antigen stimulation [37]; this effect was dependent on the release of TNF-α, and leukotriene B(4) [38] and mediated by CCL3 [39] . Increased neutrophil recruitment was also observed when SGS immunization was conducted in the ear dermis: SGS challenge led to development of an inflammatory reaction characterized by the presence of numerous neutrophils, confirming previously published results [10]. Similarly, exposure of mice to the bites of uninfected *L. longipalpis*, the vector of *L. chagasi*, induced an analogous effect [12]. In addition, it has been shown that PSG, the proteophosphoglycan-rich gel secreted by *L. mexicana*, also leads to potent neutrophil and macrophage recruitment [40].

In naïve mice, sand fly saliva [4], [5], [41]–[43] and fPPG, a component in PSG [44],favor the initial establishment of *Leishmania* infection. In naïve mice, pouch stimulation with *L. braziliensis*+SGS was unable to alter the cellular recruitment induced by *L. braziliensis* alone (Figure 5A), as opposed to previous studies conducted with *L. longipalpis* SGS+*L. chagasi* [20] or with *L. major*+*L. longipalpis* SGS [45]; however, pouch stimulation with Lb+SGS induced significant upregulation in the expression of CCL2, CXCL10, TNF-α, and IL-10 (Figure 6A). Accordingly, experimental infection with *L. braziliensis* leads to increased leukocyte recruitment, CCL2 and CXCL10 expression [46], and production of IL-10 [21]. More recently, increase CXCL10 and IL-10 expression were observed upon infection of human monocytes with *L. braziliensis* [47]. Therefore, we can suggest that, although presence of sand fly saliva does not add to the cellular recruitment induced by *L. braziliensis*, salivary antigens modulate the microenvironment, which may favor parasite establishment as previously suggested [48]. Here we were unable to determine parasite load in cellular exudates obtained from stimulated pouches; however, earlier work from our group also showed that pre-treatment of human monocytes with *L. intermedia* SGS followed by *L. braziliensis* infection led to a significant increase in TNF-α production without significant augmentation in the parasite load [18].

Pre-exposure to *L. longipalpis* [9] or *P. papatasi* saliva [5] or to bites from uninfected *P. papatasi* [49] results in protection against leishmaniasis; however, pre-exposure to *L. intermedia* saliva does not generate a protective effect upon a challenge infection with *L. braziliensis*+*L. intermedia* SGS [10] although SGS immunized mice do show a significantly lower initial parasite burden after challenge with *L. braziliensis*+SGS. We hypothesized that this early control in parasite load could be exerted by inflammatory cells (mono and polymorphonuclear cells) that are recruited following stimulation with saliva [10]. Indeed, the results herein show that SGS-immune mice displayed increased leukocyte recruitment, with a marked neutrophil influx (Figure 3) and a similar finding was observed upon inoculation of Lb+SGS (Figure 5). We have recently shown that macrophages and neutrophils collaborate towards *L. braziliensis* elimination from infected macrophages [50]. Therefore, the current results support our previous hypothesis that an initial inflammatory environment may account for the early control of parasite load in SGS-immunized mice upon challenge with Lb+SGS. This control, however, is limited and *L. braziliensis* multiplication is later on observed, probably resulting from the pathogen favorable immune response (lower IFN-γ to IL-4 ratio) developed in SGS-immunized mice [10]. Indeed, in the present work, SGS-immunized mice stimulated with Lb+SGS showed decreased CXCL10 expression paralleled with an increased IL-10 expression. Presence of CXCL10 is seen in many Th1-type inflammatory diseases, where it is thought to play an important role in recruiting activated T cells into sites of tissue inflammation [51]. IL-10, on the contrary, is associated with a non-healing *L. major* infection [52] and *L. major* persistence [53]. Consequently, lack of CXCL10 and presence of IL-10 may create a de-activating environment, favoring *L. braziliensis* expansion in the context of SGS-immunized mice.

We cannot exclude that the increased neutrophil recruitment observed in SGS-immunized mice may also be relevant to the “Trojan horse” model, as documented for *L. major* infection [29], in which parasites within neutrophils are silently transferred to macrophages and successfully establish infection. Indeed, the early influx and persistence of neutrophils after sand fly transmission of *L. major* appears critical for the development of cutaneous disease [27]. Additionally, *L. major* internalization delays the neutrophil apoptotic death program and induces CCL4 release, which recruits macrophages to the infection site [29], [54]. Indeed, increased CCL4 expression was observed upon inoculation of Lb+SGS.

Collectively, our data show that in naïve mice, inoculation of *L. intermedia* saliva plus *L. braziliensis* modifies the initial inflammatory environment as seen by increased neutrophil recruitment and IL-10 and TNF-α expression. Crucially, in mice sensitized with *L. intermedia* saliva and stimulated with *L. braziliensis*, these initial events are further modulated, as seen by a specific decrease in CXCL10 and a persistently increased IL-10 expression. We can speculate that the resulting effects leads to the higher disease burden as previously documented [10]. This study again shows important effects of the *L. intermedia* sand fly and *L. braziliensis* interaction. More important, it emphasizes how the immune response to sand fly may exert an under-appreciated role in endemic areas. We are currently characterizing *L. intermedia* salivary antigens to further identify the components that may induce the effects described here.
